# Supplementary material for: Apigenin Increases SHIP-1 Expression, Promotes Tumoricidal Macrophages and Anti-Tumor Immune Responses in Murine Pancreatic Cancer
Source: Cancers (Basel). 2020 Dec 4;12(12):3631. doi: 10.3390/cancers12123631 (PMC7761852; doi:10.3390/cancers12123631)
Supplement: Supplementary file 1 [file cancers-12-03631-s001.pdf]

Supplementary Materials: Apigenin Increases SHIP-1 Expression, Promotes Tumoricidal Macrophages and Anti-Tumor Immune Responses in Murine Pancreatic Cancer

Krystal Villalobos-Ayala, Ivannie Ortiz Rivera, Ciara Alvarez, Kazim Husain, DeVon DeLoach, Gerald Krystal, Margaret L Hibbs, Kun Jiang, Tomar Ghansah

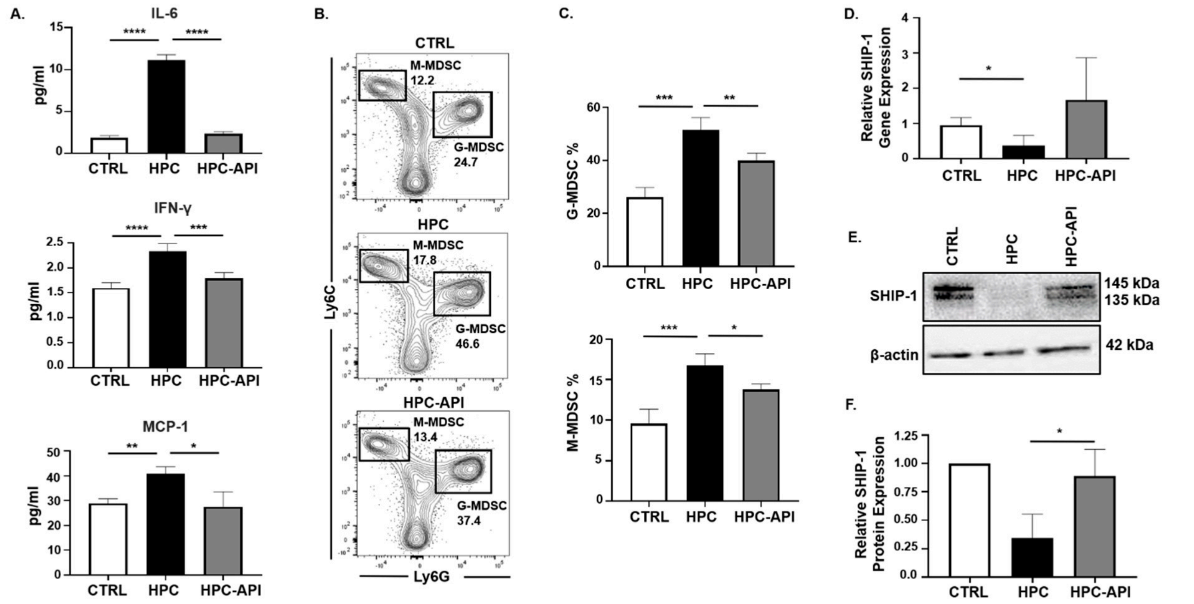

Figure S1. API reduces inflammation, MDSC percentages and increases SHIP-1 in HPC mice. (**A.**) Cytokine and chemokine profiles from the serum of CTRL, HPC and HPC-API mice as measured by Cytometric Bead Array and flow cytometry. (**B.** and **C.**) Flow cytometry analysis and representative quantification of MDSC subsets, G-MDSC (CD11b^{+}Ly6C$^{+/-}$Ly6G^{+}) and M-MDSC (CD11b^{+}Ly6G^{-}Ly6C^{+}), in the spleen from CTRL, HPC and HPC-API treated mice. (**D.**) Relative quantification of SHIP-1 gene expression in the whole splenocytes of CTRL, HPC and HPC-API mice. (**E.** and **F.**) Western blot analysis and representative quantification of normalized densitometry ratios of SHIP-1 protein levels in whole lysates from CTRL, HPC and HPC-API mice splenocytes. Data are represented as the mean ± S.D. of CTRL (n = 3-4), HPC (n = 3-6) and HPC-API (n = 3-4) mice. *$p < 0.05$; **$p < 0.01$; ***$p < 0.001$; ****$p < 0.0001$ (by two-tailed t test).

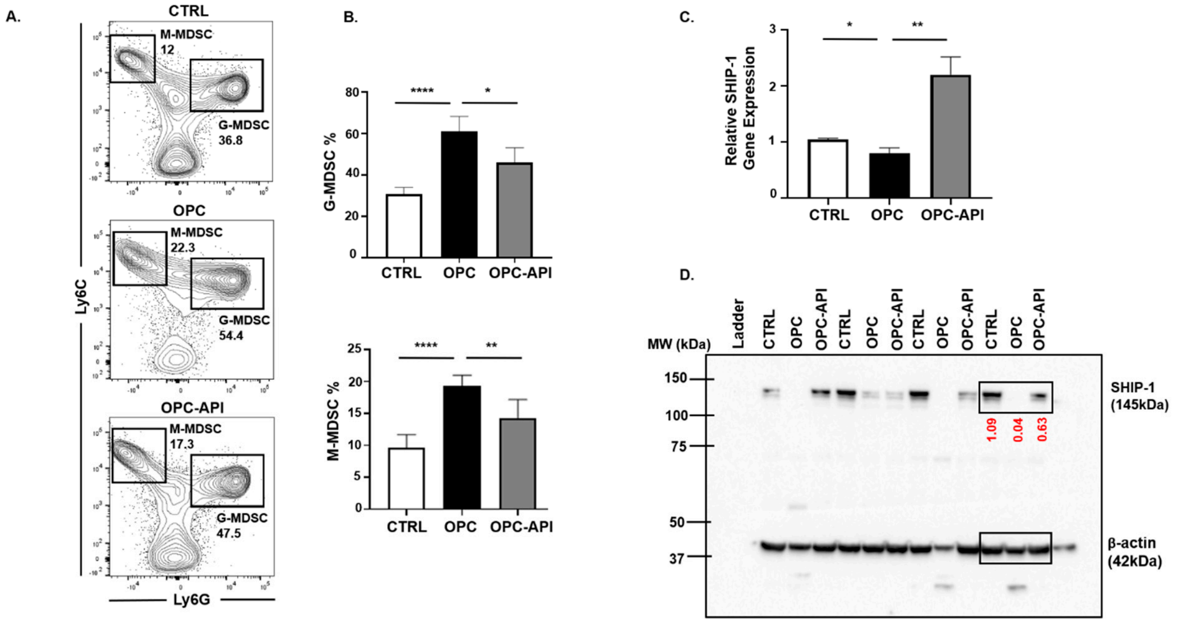

Figure S2. API reduces splenic MDSC subsets and increases SHIP-1 expression in OPC mice. (**A.** and **B.**) Flow cytometry analysis and representative quantification of MDSC subsets, G-MDSC (CD11b$^+$Ly6C$^{+/-}$Ly6G$^+$) and M-MDSC (CD11b$^+$Ly6G$^-$Ly6C$^+$), from spleens of CTRL, OPC and OPC-API treated mice. (**C.**) Relative quantification of SHIP-1 gene expression in the whole splenocytes of CTRL, OPC and OPC-API mice. (**D.**) Uncropped western blot image of SHIP-1 and β-actin. Bands that are bordered were used in Figure 2C and normalized densitometry ratio denoted in red (divided by β-actin). Data are represented as the mean ± S.D. of CTRL (n = 3-8), OPC (n = 3-5) and OPC-API (n = 3-7) mice. *p < 0.05; **p < 0.01; ****p < 0.0001 (by two-tailed t test).

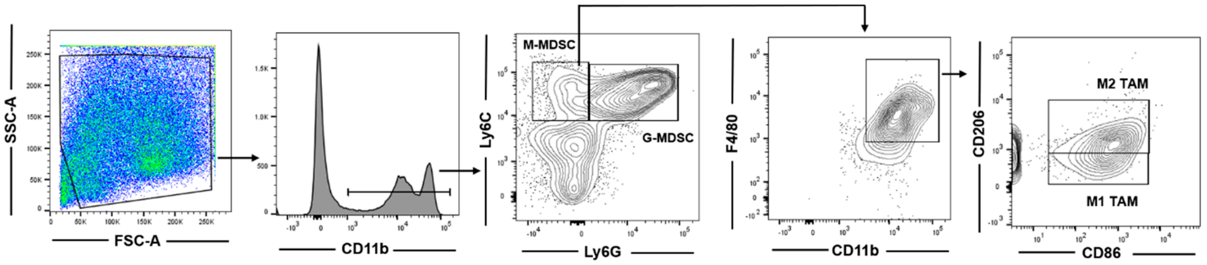

Figure S3. Gating strategy used to define MDSC and TAM subsets in all murine PC models used in this study. MDSC subsets were defined as, G-MDSC (CD11b$^+$Ly6C$^{+/-}$Ly6G$^+$) and M-MDSC (CD11b$^+$Ly6G$^-$Ly6C$^+$), and TAM subsets were defined as, M1-like TAM (CD11b$^+$Ly6C$^+$Ly6G$^-$F4/80$^+$CD206$^-$CD86$^+$) and M2-like TAM (CD11b$^+$Ly6C$^+$Ly6G$^-$F4/80$^+$CD86$^-$CD206$^+$).

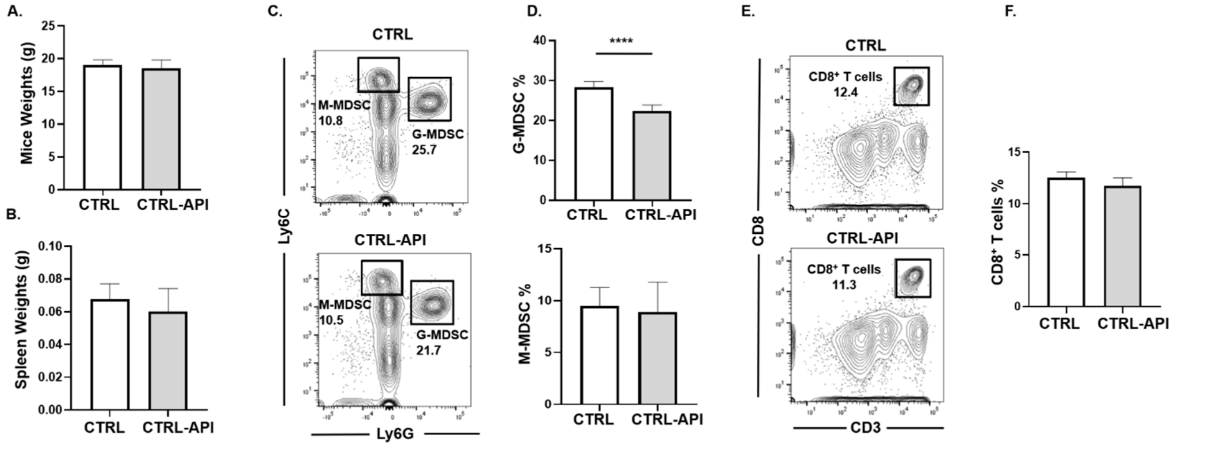

Figure S4. It appears that API treatment has minimal immunomodulatory effects on tumor-free C57BL/6 mice. (**A.** and **B.**) Mice and spleens weights of CTRL and CTRL-API mice at the endpoint of the study. (**C.** and **D.**) Flow cytometry analysis and representative quantification of MDSC subsets, G-MDSC (CD11b$^+$Ly6C$^{+/-}$Ly6G$^+$) and M-MDSC (CD11b$^+$Ly6G$^-$Ly6C$^+$), from splenocytes of CTRL and CTRL-API treated mice. (**E.** and **F.**) Flow cytometry analysis and representative quantification of CD8+ T cells (CD3$^+$CD8$^+$) from splenocytes of CTRL and CTRL-API treated mice. Data are representative as the mean ± S.D. of CTRL (n = 4-7) and CTRL-API (n = 4-5) mice. ****$p < 0.0001$ (by two-tailed t test).

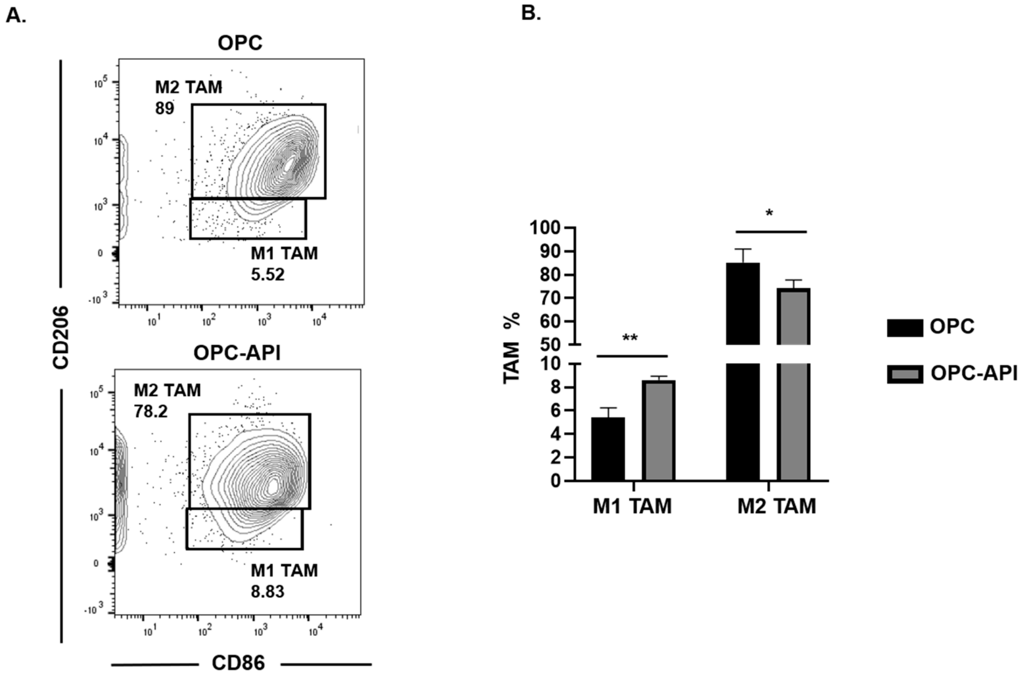

Figure S5. API increases Ly6C^{Low} M1-like TAM and decreases Ly6C^{Low} M2-like TAM in the TME of OPC mice. (**A.** and **B.**) Flow cytometry analysis and quantification of Ly6C^{Low} TAM subsets, M1-like TAM (CD11b$^+$Ly6C^{low}Ly6G$^-$F4/80$^+$CD206$^-$CD86$^+$) and M2-like TAM (CD11b$^+$Ly6C^{low}Ly6G$^-$F4/80$^+$CD86$^-$CD206$^+$) in

whole pancreatic tumors from OPC and OPC-API mice. Data are represented as the mean ± S.D. of OPC (n = 3) and OPC-API (n = 3) mice. *$p < 0.05$; **$p < 0.01$ (by two-tailed t test).

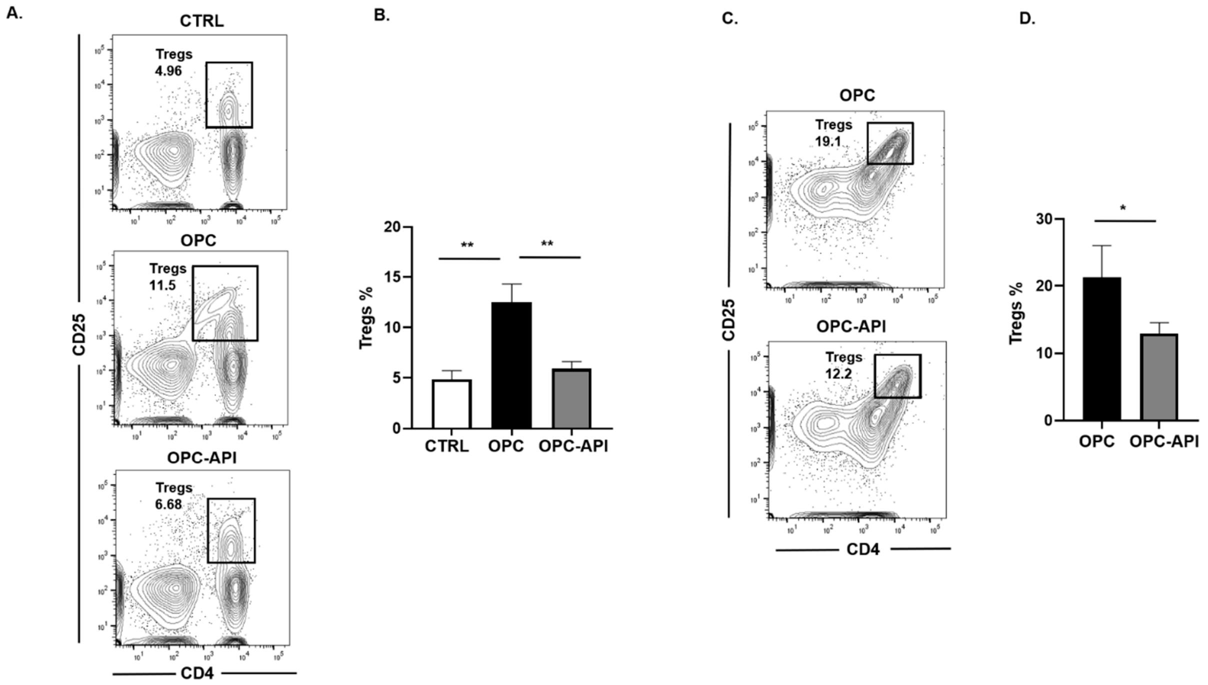

Figure S6. API decreases Treg percentages in the spleen and TME of OPC mice. Flow cytometry analysis and representative quantification of Tregs ($CD3^+CD4^+CD25^+$) in the spleen (**A.** and **B.**) and pancreatic tumor (**C.** and **D.**) of CTRL, OPC, and OPC-API mice. Data are represented as the mean ± S.D. of CTRL (n = 3), OPC (n = 3) and OPC-API (n = 3) mice. *$p < 0.05$; **$p < 0.01$ (by two-tailed t test).

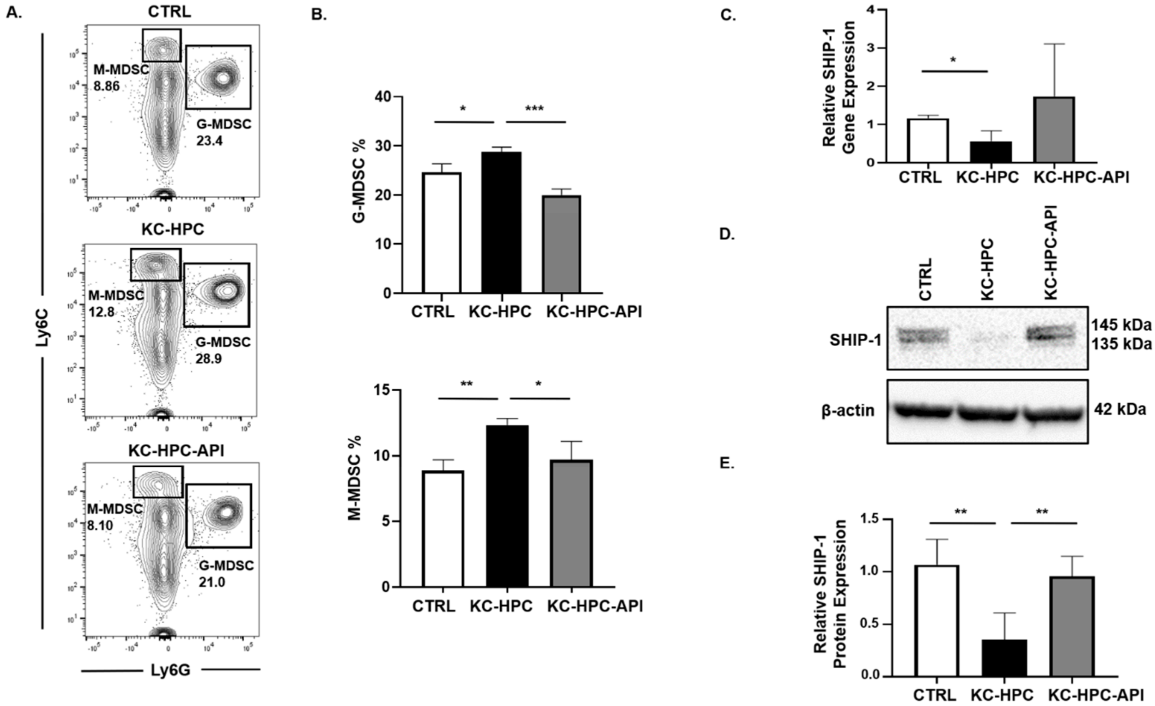

Figure S7. API reduces proportion of splenic MDSC subsets and increases SHIP-1 expression in KC-HPC mice. (**A.** and **B.**) Flow cytometry analysis and representative quantification of MDSC subsets, G-MDSC (CD11b^{+}Ly6C$^{+/-}$Ly6G^{+}) and M-MDSC (CD11b^{+}Ly6G^{-}Ly6C^{+}), from spleens of CTRL, KC-HPC and KC-HPC-API treated mice. (**C.**) Relative quantification of SHIP-1 gene expression in the whole splenocytes of CTRL, KC-HPC and KC-HPC-API mice. (**D.** and **E.**) Western blot analysis and representative quantification of normalized densitometry ratios of SHIP-1 protein levels in whole cell lysates from CTRL, KC-HPC and KC-HPC-API mice splenocytes. Data are represented as the mean ± S.D. of CTRL (n = 3), KC-HPC (n = 3-4) and KC-HPC-API (n = 3-4) mice. *$p < 0.05$; **$p < 0.01$; ***$p < 0.001$ (by two-tailed *t* test).

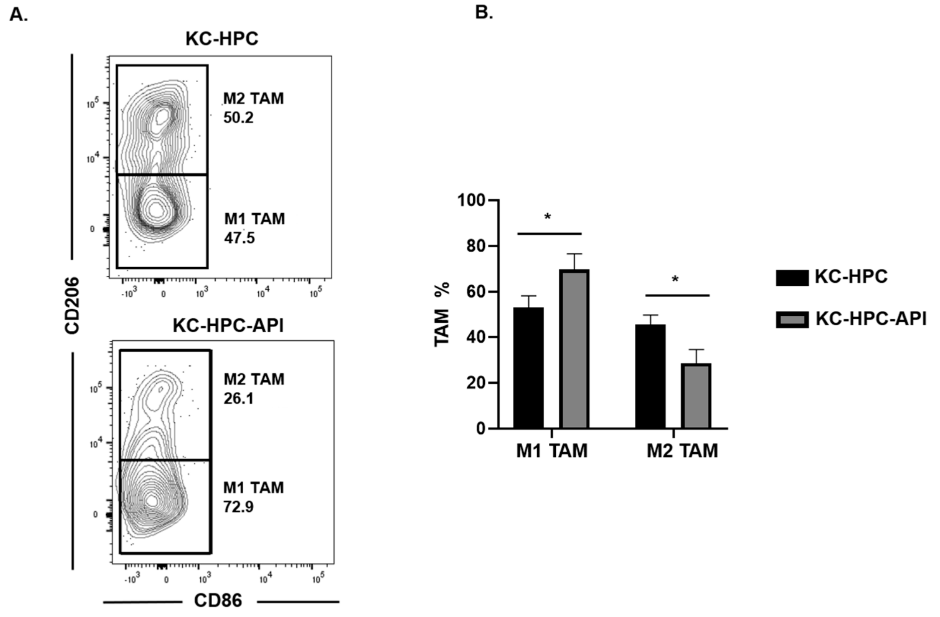

Figure S8. API increases Ly6C^{Low} M1-like TAM and decreases Ly6C^{Low} M2-like TAM in the TME of KC-HPC mice. (**A.** and **B.**) Flow cytometry analysis and quantification of Ly6C^{Low} TAM subsets, M1-like TAM (CD11b$^+$Ly6C^{low}Ly6G$^-$F4/80$^+$CD206$^-$CD86$^+$) and M2-like TAM (CD11b$^+$Ly6C^{low}Ly6G$^-$F4/80$^+$CD86$^-$CD206$^+$) in whole tumors from KC-HPC and KC-HPC-API mice. Data are represented as the mean ± S.D. of KC-HPC (n = 3) and KC-HPC-API (n = 3) mice. *$p < 0.05$ (by two-tailed *t* test).

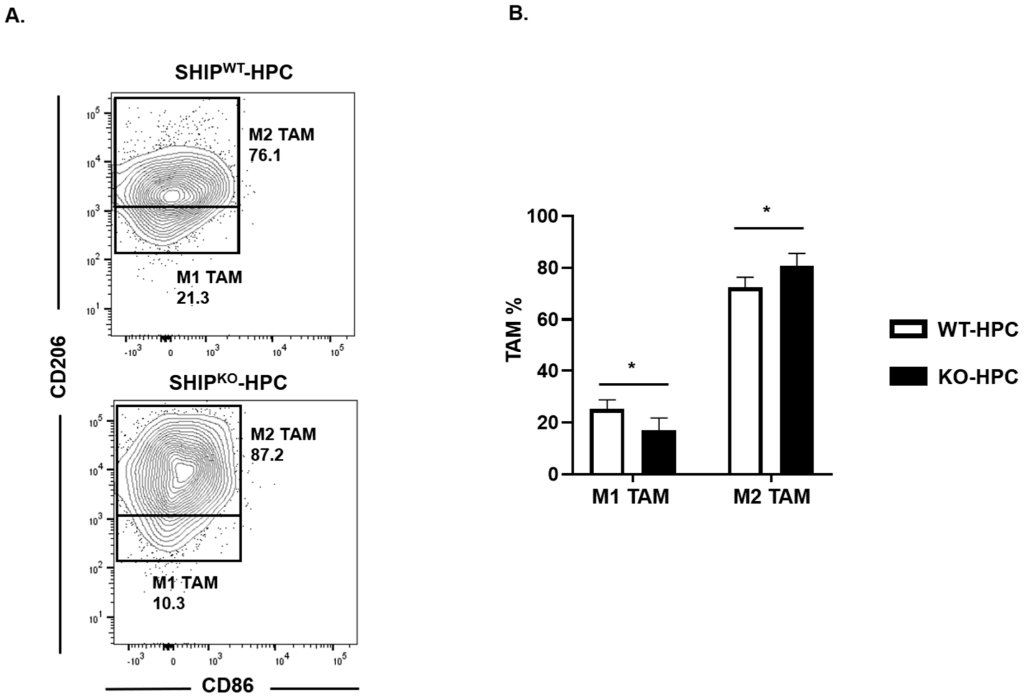

Figure S9. SHIP-1 expression controls the development of Ly6C^{Low} M1-like TAM in HPC. (A. and B.) Flow cytometry analysis and quantification of Ly6C^{Low} TAM subsets, M1-like TAM (CD11b$^+$Ly6C^{low}Ly6G$^-$F4/80$^+$CD206$^-$CD86$^+$) and M2-like TAM (CD11b$^+$Ly6C^{low}Ly6G$^-$F4/80$^+$CD86$^-$CD206$^+$) in the whole tumors of SHIP^{WT}-HPC and SHIP^{KO}-HPC mice. SHIP^{WT}-HPC (WT-HPC) and SHIP^{KO}-HPC (KO-HPC). Data are represented as the mean ± S.D. of SHIP^{WT}-HPC (n = 3) and SHIP^{KO}-HPC (n = 5) mice. *$p < 0.05$ (by two-tailed t test).

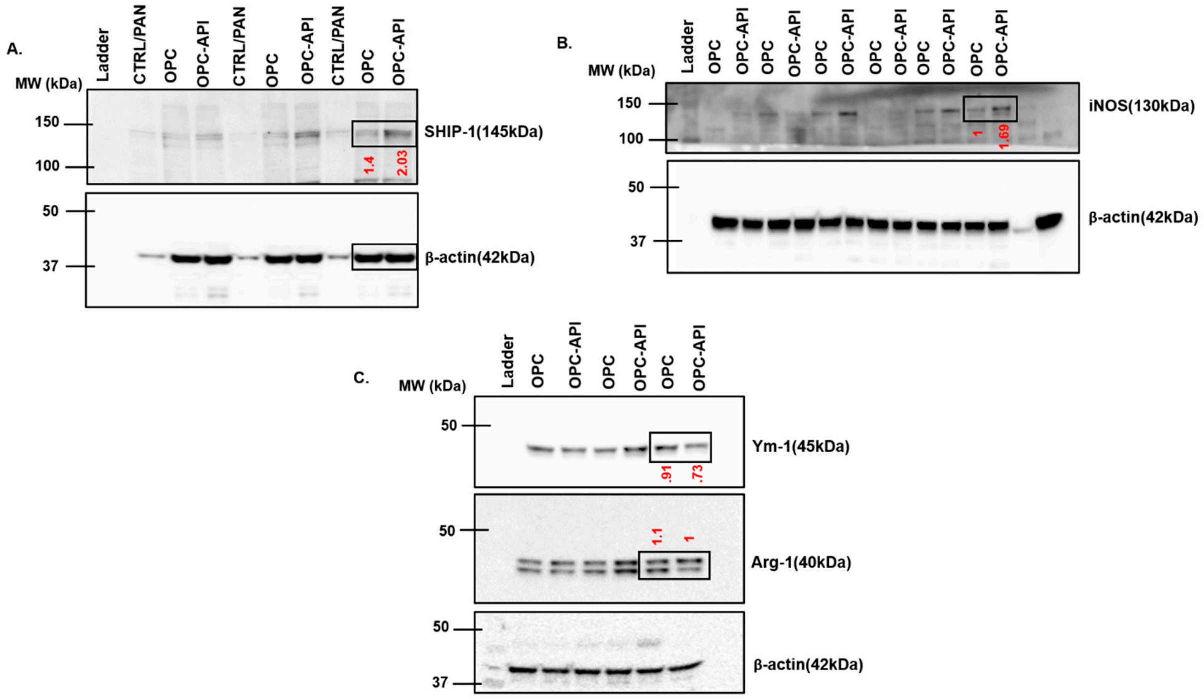

Figure S10. Uncropped western blot images from Figure 6D. Uncropped western blot images of SHIP-1 (**A.**), iNOS (**B.**), Ym-1 (**C.**), Arg-1 (**C.**) and β-actin. Bands that are bordered were used in Figure 6D and normalized densitometry ratio denoted in red (divided by β-actin).

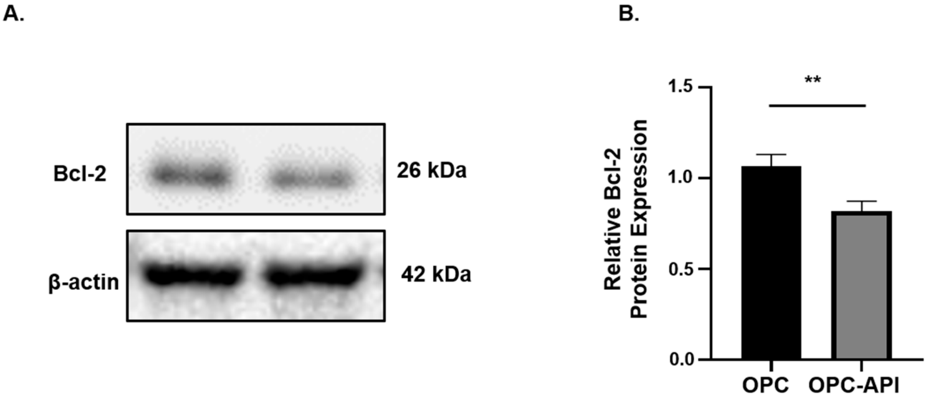

Figure S11. API decreases the anti-apoptotic protein, Bcl-2, in the TME of OPC mice. (**A.** and **B.**) Western blot analysis and quantification of normalized densitometry ratios of Bcl-2 protein in whole cell lysates from vehicle treated OPC mice and API treated OPC mice tumors. Data are represented as the mean ± S.D. of OPC (n = 3) and OPC-API (n = 3) mice. **$p < 0.01$ (by two-tailed t test).

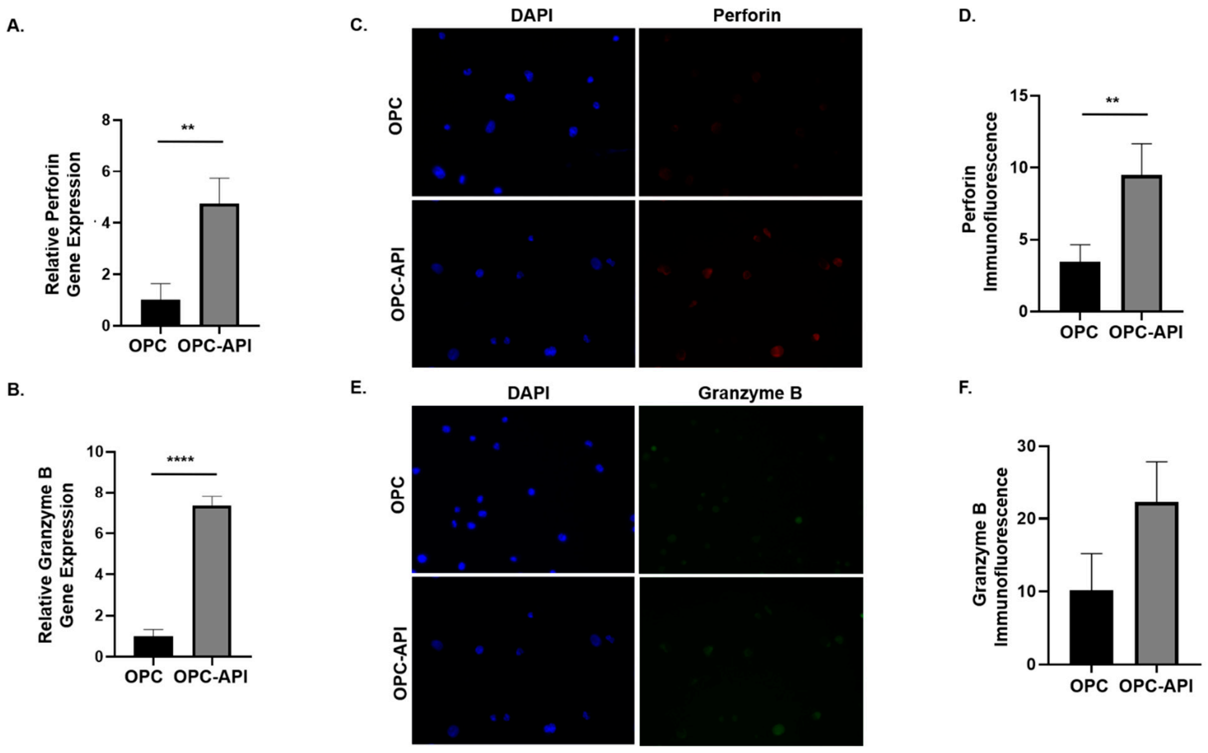

Figure S12. API increases Perforin and Granzyme B expression in the TME of OPC mice. (**A.** and **B.**) Relative quantification of perforin and granzyme B gene expression in the whole pancreatic tumors of OPC and OPC-API mice. Fluorescent microscopy images (40 ×) and representative quantification of perforin (red) (**C.** and **D.**) and granzyme B (green) (**E.** and **F.**) immunofluorescences from the pancreatic tumor of OPC and OPC-API mice. Nuclear DNA appears as blue (DAPI). Data are representative as the mean ± S.D. of OPC (n = 3) and OPC-API (n = 3-4) mice. $**p < 0.01$; $****p < 0.0001$ (by two-tailed t test).

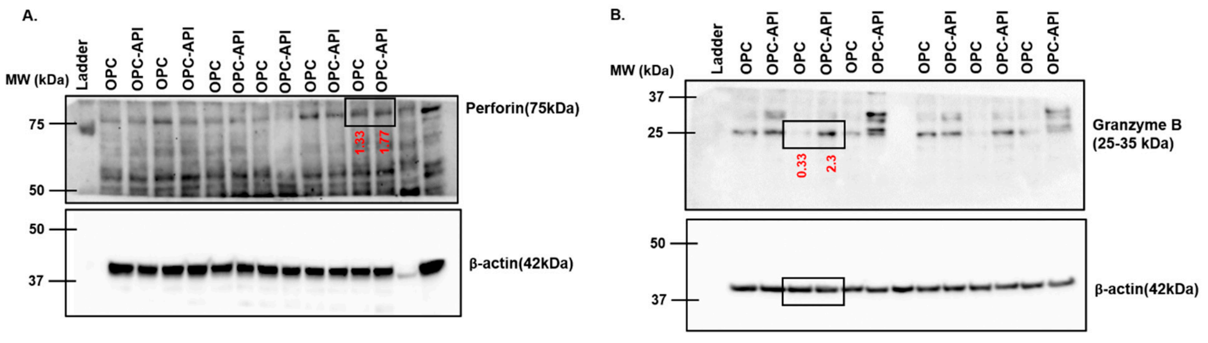

Figure S13. Uncropped western blot images from Figure 7G. Uncropped western blot images of Perforin (**A.**), Granzyme B (**B.**) and β-actin. Bands that are bordered were used in Figure 7G and normalized densitometry ratio denoted in red (divided by β-actin).
